# Microwave bio-sensor based on symmetrical split ring resonator with spurline filters for therapeutic goods detection

**DOI:** 10.1371/journal.pone.0185122

**Published:** 2017-09-21

**Authors:** Rammah A. Alahnomi, Z. Zakaria, E. Ruslan, S. R. Ab Rashid, Amyrul Azuan Mohd Bahar, Azizah Shaaban

**Affiliations:** 1 Center for Telecommunication Research and Innovation (CeTRI), Universiti Teknikal Malaysia Melaka (UTeM), Hang Tuah Jaya, Durian Tunggal, Melaka, Malaysia; 2 Faculty of Technology Engineering (FTK), Universiti Teknikal Malaysia Melaka (UTeM), Hang Tuah Jaya, Durian Tunggal, Melaka, Malaysia; 3 Department of Engineering Materials, Faculty of Manufacturing Engineering, Universiti Teknikal Malaysia Melaka (UTeM), Hang Tuah Jaya, Durian Tunggal, Melaka, Malaysia; Leibniz-Institut fur Pflanzengenetik und Kulturpflanzenforschung Gatersleben, GERMANY

## Abstract

A novel symmetrical split ring resonator (SSRR) based microwave sensor with spurline filters for detecting and characterizing the properties of solid materials has been developed. Due to the weak perturbation in the interaction of material under test (MUT) and planar microwave sensor, spurline filters were embedded to the SSRR microwave sensor which effectively enhanced Q-factor with suppressing the undesired harmonic frequency. The spurline filter structures force the presented sensor to resonate at a fundamental frequency of 2.2 GHz with the capabilities of suppressing rejected harmonic frequency and miniaturization in circuit size. A wide bandwidth rejection is achieved by using double spurlines filters with high Q-factor achievement (up to 652.94) compared to single spurline filter. The new SSRR sensor with spurline filters displayed desired properties such as high sensitivity, accuracy, and performance with a 1.3% typical percentage error in the measurement results. Furthermore, the sensor has been successfully applied for detecting and characterizing solid materials (such as Roger 5880, Roger 4350, and FR4) and evidently demonstrated that it can suppress the harmonic frequency effectively. This novel design with harmonic suppression is useful for various applications such as food industry (meat, fruit, vegetables), biological medicine (derived from proteins and other substances produced by the body), and Therapeutic goods (antiseptics, vitamins, anti-psychotics, and other medicines).

## Introduction

In the last few years, there has been an explosive growth of interest in microwave sensors for various technology developments such as characterizing the properties of the materials with their structural composition analysis. These properties are directly characterized and determined based on the sensitivity of the microwave resonator sensors. The composition of materials, moisture or water content of the material under test (MUT) carry useful information and electrical properties of these materials depend on their properties. Thus, the quality control in material science, in food industry, bio-sensing can be conducted based on sensing electrical properties of materials [1–4]. Conventionally, coaxial cavity, waveguide, dielectric resonator sensors have been used for characterizing and detecting material properties. However, these sensors are often large, and expensive to build, which restricts their use in many important applications. Thus, planar resonant sensors have gained a considerable interest due to the advantages of having compactness in size, ease of fabrication, and low cost. This microwave bio-sensing technique has the potential for detecting and characterizing the properties of solid materials due to its non-invasive characteristics and penetration sensing capability.

In microwave system, the harmonic radiation causes the electromagnetic interference (EMI) which is considered as an important problem in effecting the performance of the radio frequency (RF) front-end modules [1,5]. Thus, minimizing harmonic distortion from nonlinear active components is a great challenge in modern microwave technologies. Both passive and active filters are widely used to suppress undesired signals since several decades ago. Generally, various passive components such as resonators and microstrip filters are proposed to achieve harmonic suppression. For instance, the Wilkinson power divider using 4n open stub technique for the harmonic suppression which was reported in [6]. Recently, many popular techniques are applied in order to suppress high-order harmonic in antenna, amplifier, oscillator, and coupler such as defected ground structure (DGS), electromagnetic bandgap (EBG), and periodic photonic bandgap (PBG) [7–10]. They are used due to their simplicity with an excellent filtering performance and low cost. However, many defected cells are required in all above-mentioned techniques which result in large circuit size and more insertion loss. So, the size reduction of the single asymmetric DGS was excellent for the harmonic suppression of second and third harmonic simultaneously [11]. The DGS, PBG, and EBG require accurate position calibration due to an etching process on a backside ground plane, which improves time-consumption and difficulty in machining. However, the problems of these techniques are allowing significant backside radiation which disturbs the radiation characteristic of the RF front-end module such as microstrip antennas. Furthermore, increasing the design complexity due to the requirement of fabricating additional structure at the ground plane of the module which results high cost of the fabrication process. Among all designs of microstrip filters, spurline is considered as a simple defected structure which is embedded directly on the microstrip line of symmetrical split ring resonator (SSRR) by etching an L-shaped slot without any stubs or etching process on the backside of the ground plane and it is considered as a small structure when compared to other filters designs [12]. It can provide an excellent band-stop characteristics and moderate rejection bandwidth with its compact size [13].

In this paper, we present novel structures of planar microwave sensors based on symmetrical split ring resonator (SSRR) with a spurline filters for determining and detecting the dielectric properties in common solid materials. The spurline filter is used for producing high Q-factor with a capability to suppress the undesired harmonic spurious and undesired frequency. The spurline filters use a proper length at a suitable position on the microstrip feed-line which it can be single or double spurlines and act as band-stop filters. The significant of using new structure resonator sensor with spurline filters is for various industrial applications such as food industry, quality control, bio–sensing medicine and pharmacy.

## Method

### Design structures

**Fig 1:**
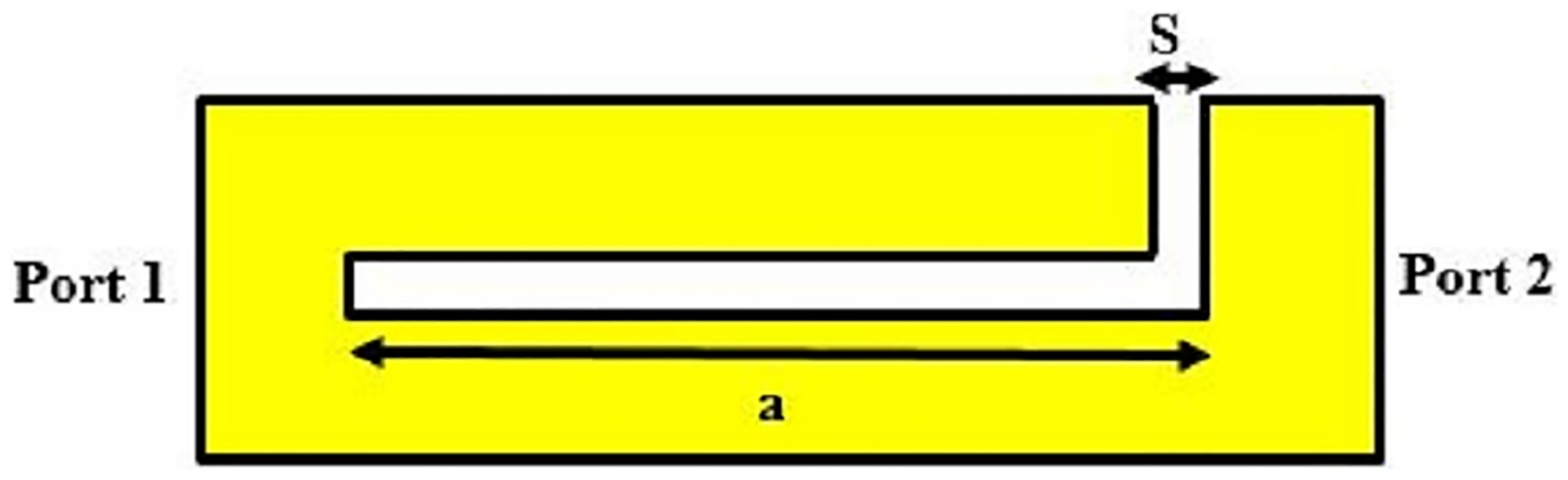
Single spurline filter configuration.

**Fig 2:**
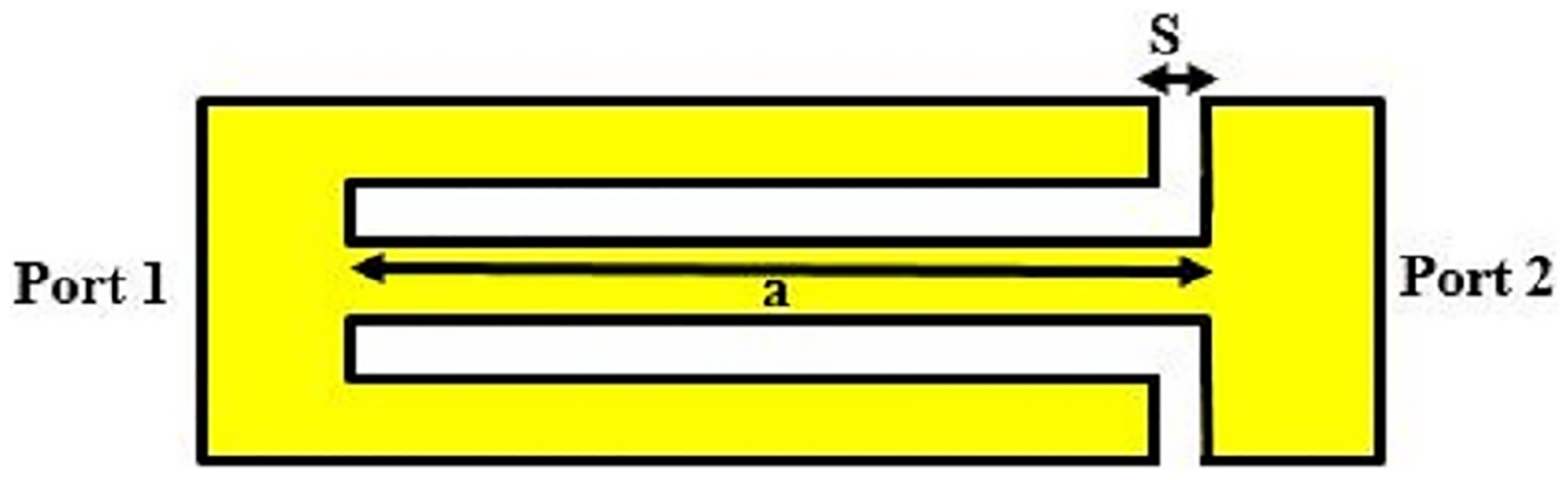
Double spurlines filter configuration.

The conventional schematic view of SSRR sensors for both single and double spurlines which work as band-stop filters is demonstrated in Figs 1 and 2, respectively. The configuration is embedded by L-shape slot with a length (a) and with a gap width (s) in the feed-line of the sensor. Generally, the slot width (s) or the gap exhibits the effect of the capacitive while the microstrip line provides an effect of the inductance. The undesired wavelength can be expressed as [13]:

$$a=\frac{\lambda_{g}}{4}$$ (1)

where *a* = the spurline length, and *λ*_*g*_ = the rejected wavelength.

By deriving Eq (20) into the frequency domain as follows [13]:

$$f_{stop}=\frac{c}{4a\sqrt{\varepsilon_{eff}}}$$ (2)

where *a* = the spurline length, *ε*_*eff*_ = the effective of substrate permittivity, *c* = speed of light (3x10^8^ *m/s*), and *f*_*stop*_ = the undesired frequency.

**Fig 3:**
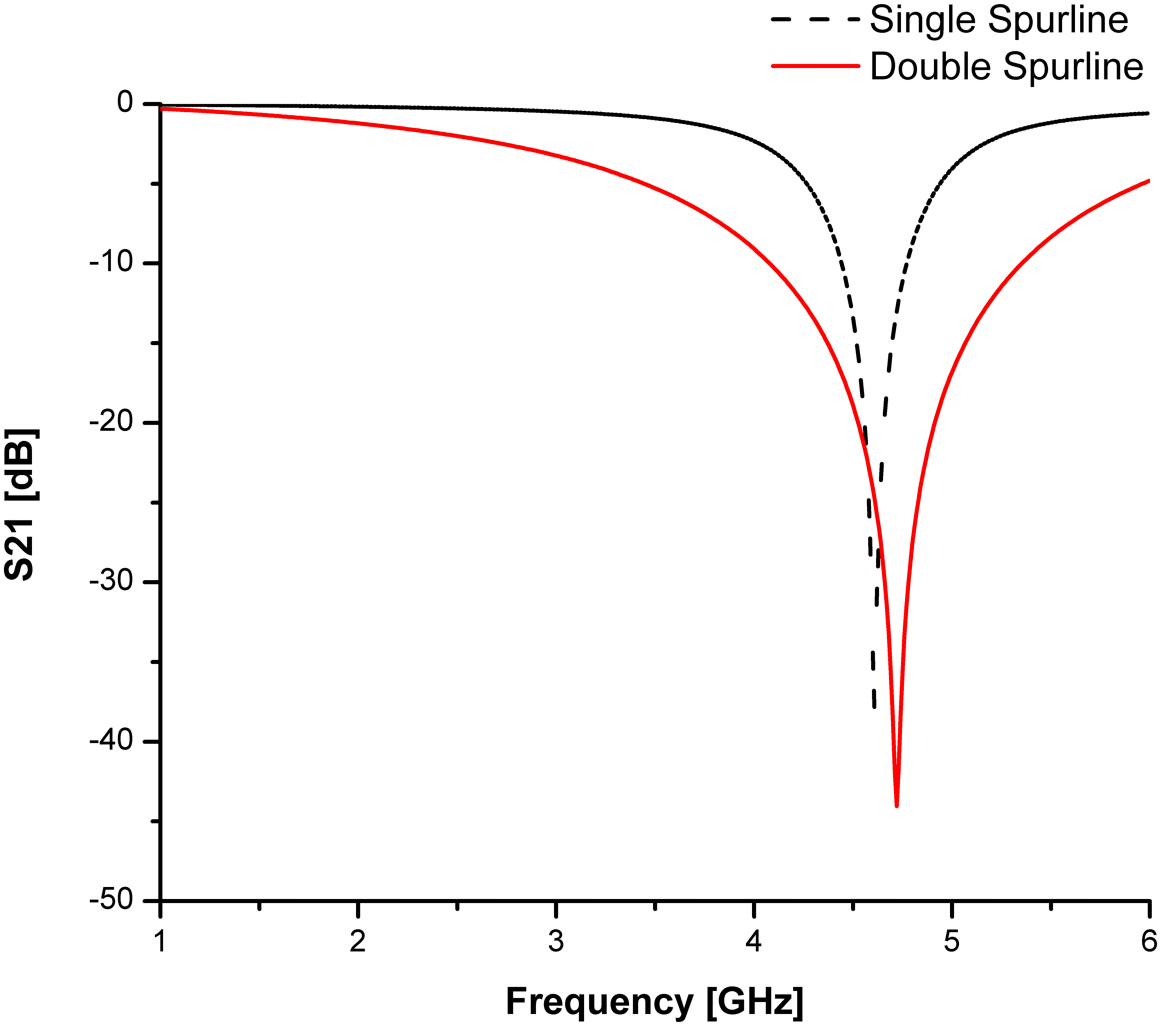
Comparison of simulated transmission coefficients for both single and double spurlines filters.

For both types of spurline filters, the Roger 5880 substrate is used with a dielectric permittivity of 2.2 (*ε*_*r*_), 0.787 *mm* thickness (*h*) and 0.0175 *mm* conductor copper (*t*). The microstrip line length is 18.85 *mm* and width of 2.5 *mm*. The spurline dimensions are *a* = 12.48 *mm* and *s* = 0.4 *mm* (refer to [14] for more details on design structures and parameters). Double spurlines is applied for obtaining a wider bandwidth rejection without increment in overall size of the circuit design. The effect of double spurlines on transmission coefficients (S21, *dB*) are studied in order to compare with a single spurline filter as indicated in Fig 3. The obtained simulation result of the rejected frequency is verified by a good agreement with the theoretical results. The stopband region at -3 dB level of the double spurlines is much more widely compared to that of single spurline. It can be clearly seen from the simulated results demonstrated in Fig 3, the double spurlines filter is having a wider bandwidth rejection and the rejection level is very deep compared to the single spurline filter. Thus, the double spurlines filter is suitable to be applied as a compact band-stop filter.

**Fig 4:**
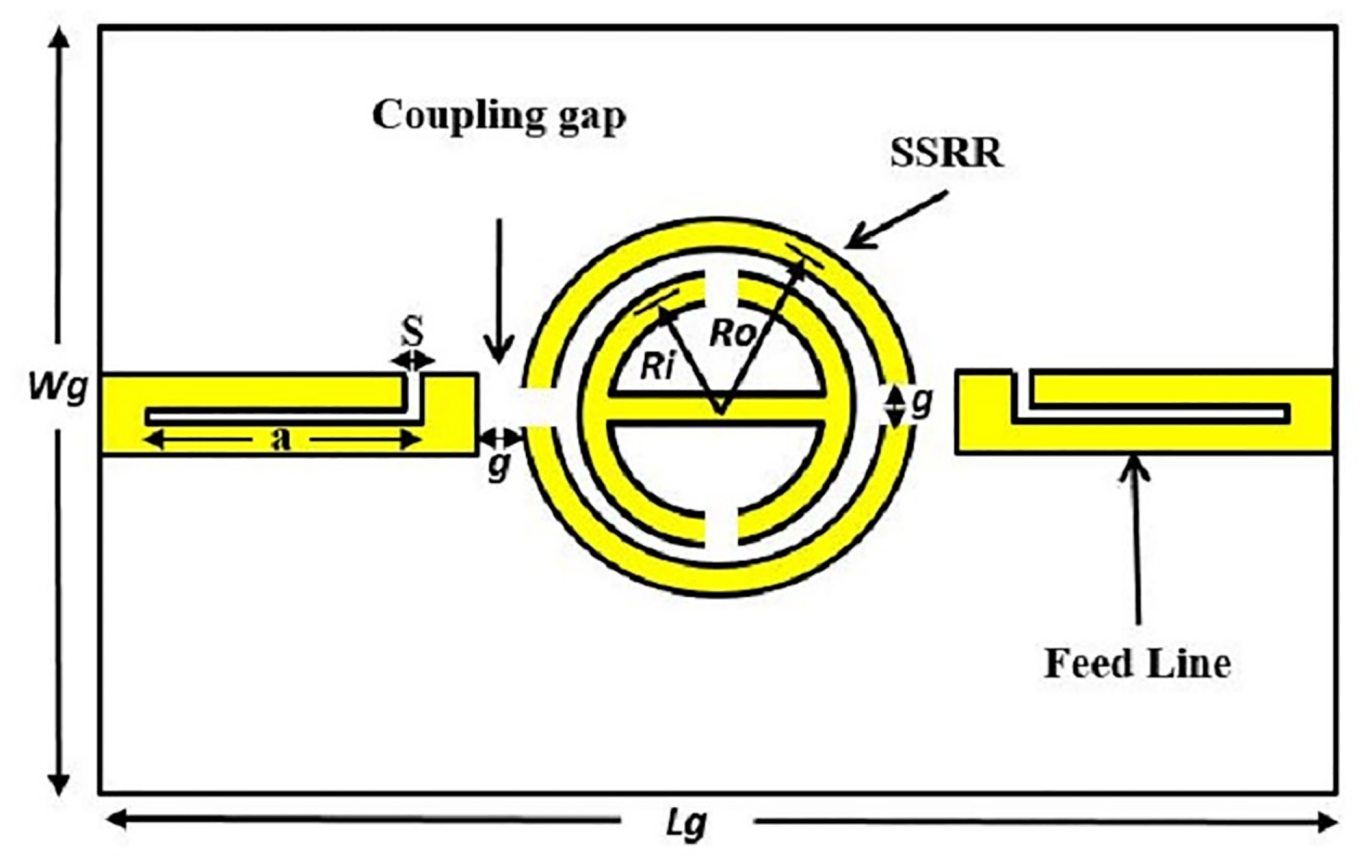
Design structure of the SSRR sensor with single spurline filter.

**Fig 5:**
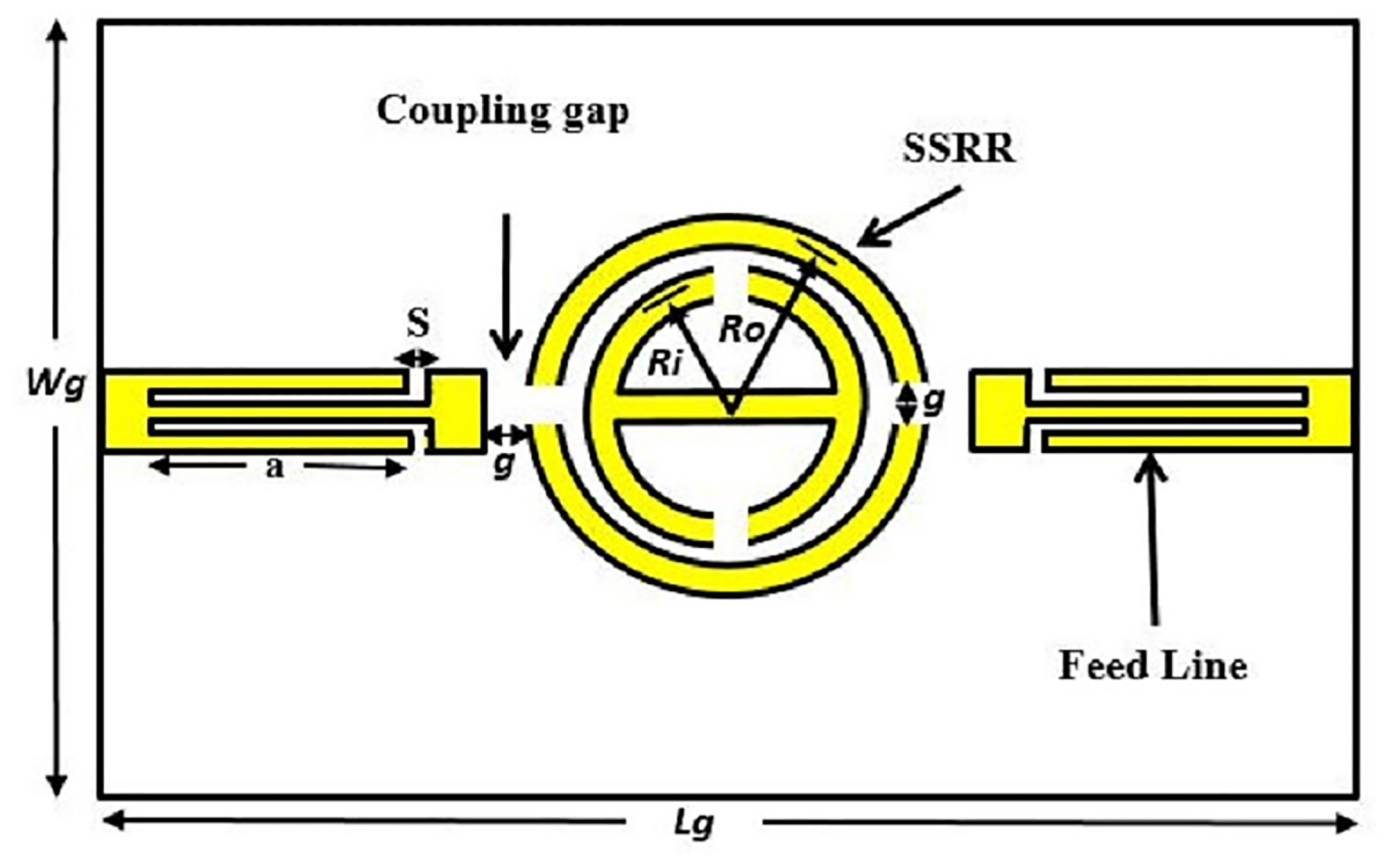
Design structure of the SSRR sensor with double spurlines filter.

These features make the proposed SSRR ring resonator compact and more suitable for microwave devices compared to other techniques. Figs 4 and 5 demonstrate the design structure of the symmetrical split ring resonator (SSRR) sensor with both single and double spurlines filters, respectively. The required specifications for microstrip planar based on symmetrical split-ring resonator (SSRR) parameters with spurlines are listed in Table 1. A total substrate length and width are 68 *mm* and 100 *mm*, respectively, and fed through with a capacitive microstrip line which satisfying the resonant conditions:

$$2\pi R=n\lambda_{g}$$ (3)

where n is the number of the mode resonance in harmonic order and R is the main ring radius.

**Table 1:** Symmetrical split-ring resonator (SSRR) with spurline design specifications.

Parameter	Design Value
Substrate: Roger RT/Duriod 5880	*ε* = 2.2
*f*	2.2 GHz
*Z*_*o*_	50 Ω
*Lg*	71.84 *mm*
*Wg*	68.3 *mm*
*h*	0.787 *mm*
*r*	15.85 *mm*
*t*	0.0175 *mm*
*w*	2.5 *mm*
*l*	34.0 *mm*
*g*	0.37 *mm*
*a*	12.76 *mm*

The resonant frequency for different modes can be expressed in Eq (4) [15].

$$f_{o}=\frac{nc}{2\pi r\sqrt{\varepsilon_{eff}}}$$ (4)

where:

*c* = 3 x 10^8^ *m/s*.

*n* = 1, 2, 3, …

*r* = Radius of the ring.

*ε*_*eff*_ = effective of substrate permittivity.

For the 50 Ω characteristic impedance *Z*_*o*_, the ratio of *w/d* can be found and the feed-line width can be expressed as [2]:

$$\frac{w}{d}=\frac{8e^{A}}{e^{2A-2}}$$ (5)

where:

$$A=\frac{Z_{o}}{60}\sqrt{\frac{\varepsilon_{r}+1}{2}}+\;\frac{\varepsilon_{r}-1}{\varepsilon_{r}+1}\left(0.23+\frac{0.11}{\varepsilon_{r}}\right)$$ (6)

The quality factor (Q-factor) can be determined using this formula:

$$Q=\frac{2f_{o}}{\Delta f}$$ (7)

where Δf is the bandwidth at +3 dB with respect to the minimal transitions.

### Electric field distributions

**Fig 6:**
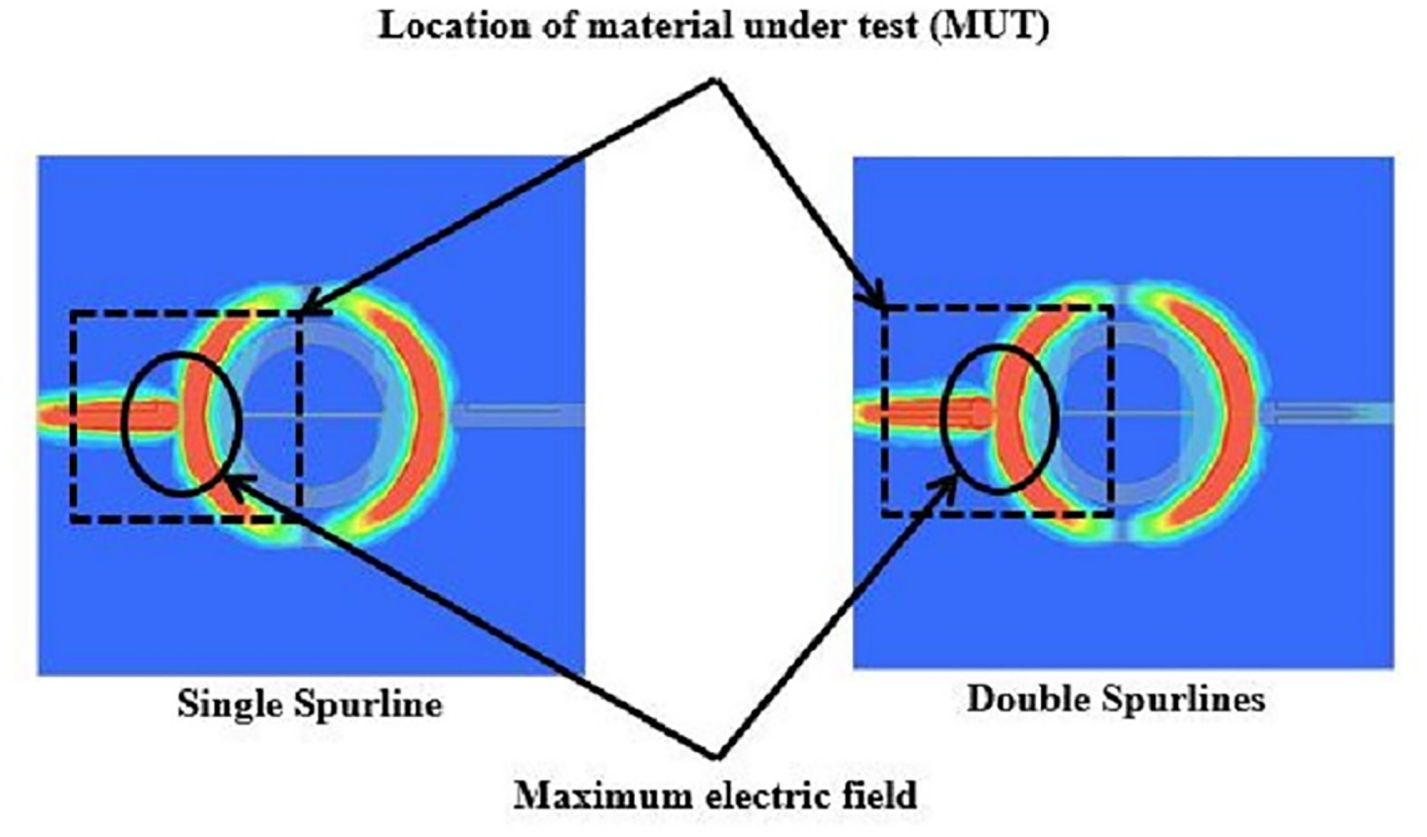
Simulated electric field on SSRR sensor with both single and double spurlines.

**Fig 7:**
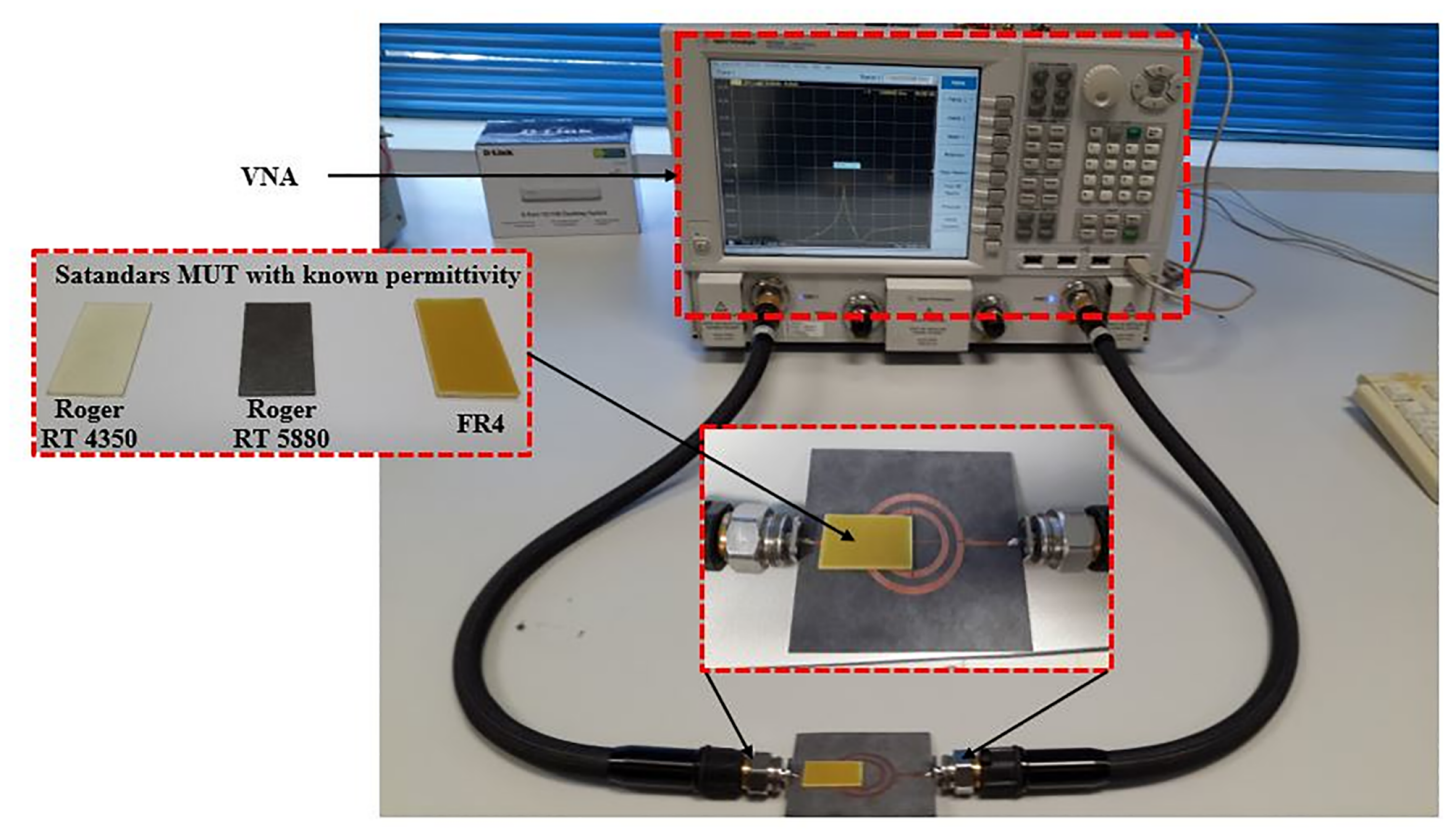
Location of measured MUT on the designed sensor.

The electric field distributions for the symmetrical split ring resonator (SSRR) with spurlines is demonstrated in Fig 6. It can be clearly seen that the coupling is electrical in nature and that maximums of several electric fields occur along the resonator length and where the feed-line excites the resonator (coupling gap between the feed-lines and ring). This is due to the independence of the azimuthal position of the feed-line that extracts microwave power. The absolute values of maximum field occur at the fundamental mode of 2.2 GHz operating frequency. The fundamental mode has a higher current distribution as indicated in Fig 6, where the red color represents the highest current distributions. The perturbation to the system can be controlled by the size and the location of the measured material. Solid material under test which may have high dielectric permittivity with high loss can be contained above the substrate of the sensor. Placing the material under test (MUT) in this manner ensures the perturbation to the system in the same amount when the measurement is made each time. A good location on the sensor where the MUT can be located on the top of the copper track on the substrate is shown in Fig 7 for symmetrical split ring resonator (SSRR) sensor with both single and double spurlines respectively. Here the electric field maximum is common in all modes. The sensitivity and accuracy of measurement are dependent on the extent of field penetration inside the material. Thus, the MUT must be located where the maximum electric field (E-field) occurs. Furthermore, Fig 6 demonstrates the possible location of the material under test (MUT) for materials measurement. It can be clearly seen that the electric field is more concentrated in the coupling gap regions (between feed-lines and ring). For this reason, it should be obvious that by locating the material under test in the maximum electric field (above the copper track of the sensor substrate) results in higher sensitivity to the above surface permittivity variations. The field perturbations in the coupling gap region are linearly proportional to the resonance shifts. A small size of the material under test (MUT) can be evaluated by utilizing the partial overlay MUT on the symmetrical split ring resonator (SSRR) sensor with single or double spurlines as illustrated in Fig 7. The partial overlay MUT provides flexibility and suitability for permittivity measurements compared to complete overlay which requires a larger size of the material under test (MUT).

## Findings and discussions

### Verification and validation of characterizing MUT

**Fig 8:**
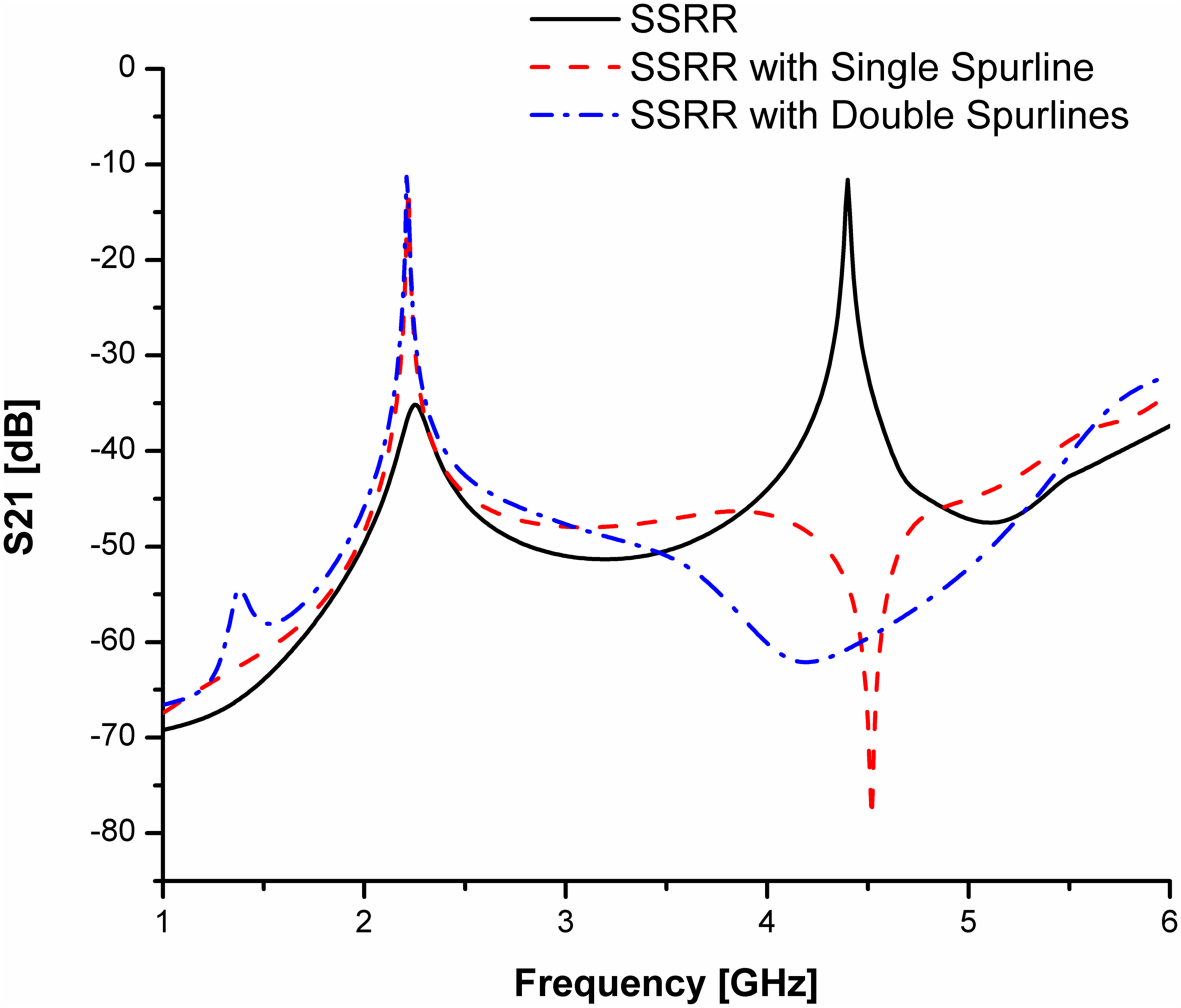
Comparison of simulated results for the designed SSRR sensors with/without spurline filters before optimization.

**Fig 9:**
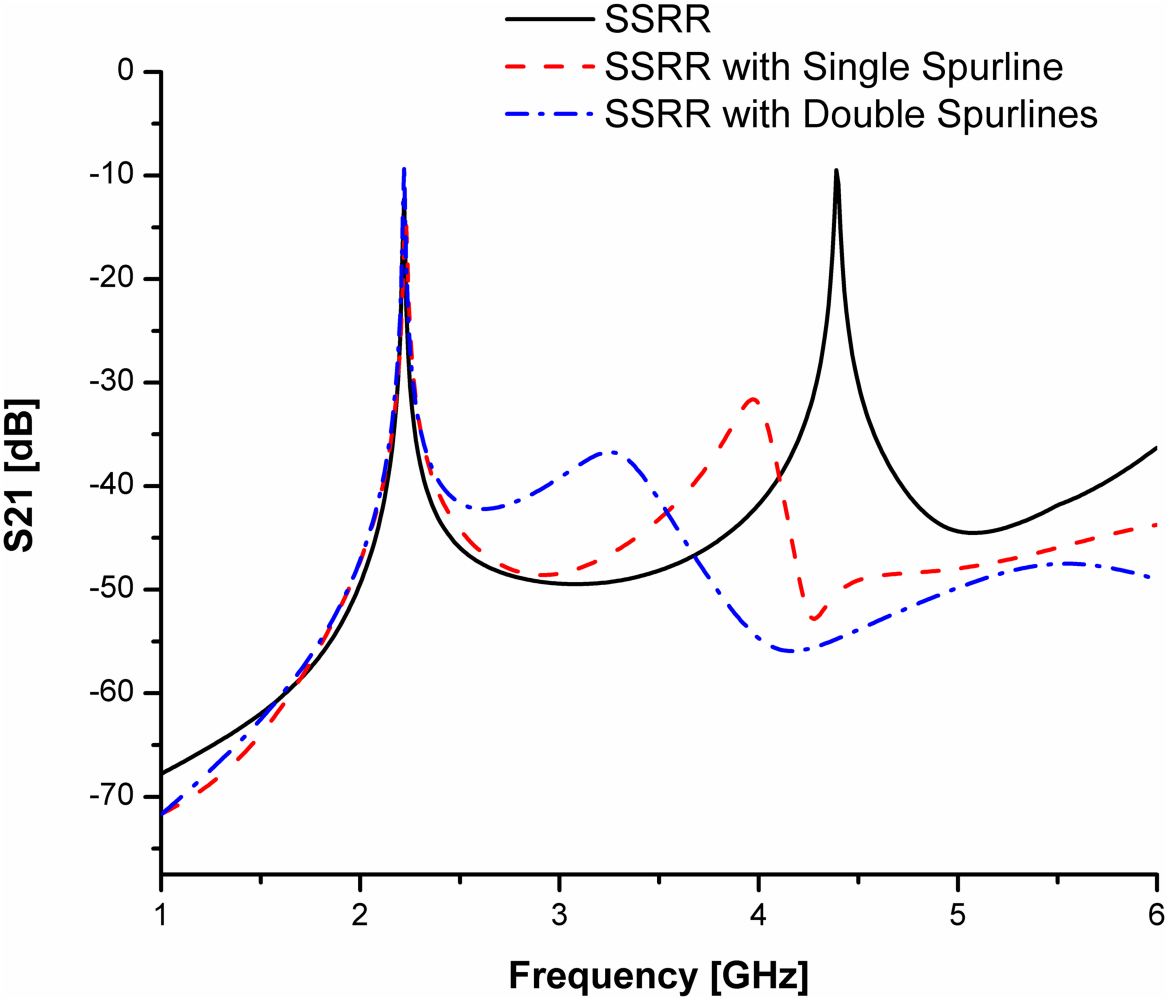
Comparison of simulated results for the designed SSRR sensors with/without spurline filters after optimization.

**Fig 10:**
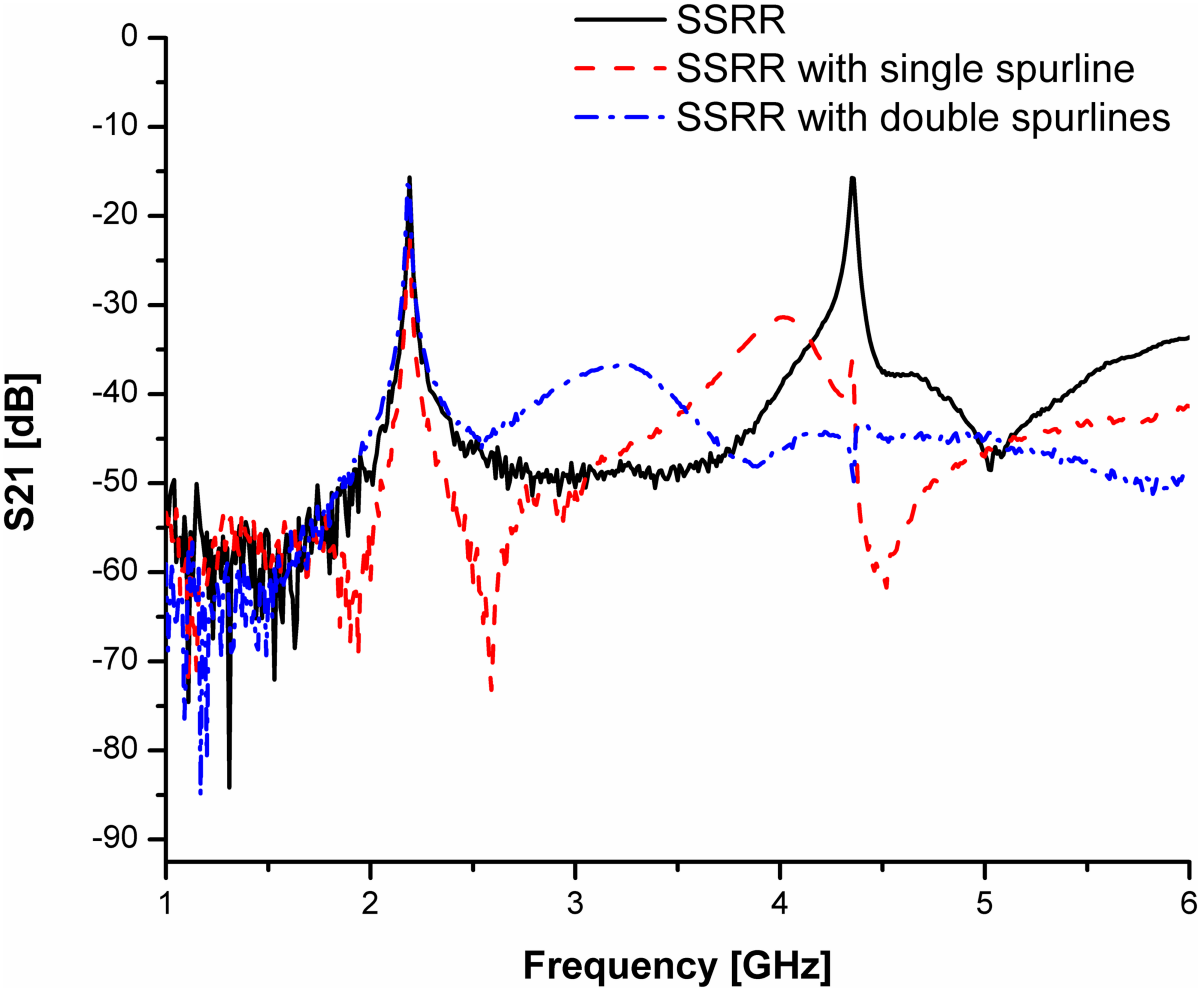
The measured permittivity of the proposed sensors in comparing with standards permittivity of MUT.

For simplicity and demonstration of concept, we will base our discussion on the comparison between the SSRR sensor and SSRR sensor with spurline filters. Figs 8 and 9 illustrate the simulation results for the designed sensors in terms of resonant frequency and transmission coefficients before and after optimization was made respectively. It can be clearly seen that the undesired harmonic frequency is totally suppressed where the SSRR sensor with double spurlines has a wider rejection bandwidth compared to single spurline. In addition, the fundamental frequency of all designed sensors is about 2.22 GHz where the rejected frequency is about 4.4 GHz. The undesired frequency obtained from simulation has a very good agreement with the theoretical expression. On the other hand, the measurement results are illustrated in Fig 10. It obviously demonstrates that the SSRR sensor with double spurlines has a sharper dip and narrower bandwidth which reveals its high Q nature of around 652.94 compared to other structures. Furthermore, results are in good agreement implying experimental results which are valid within the range of simulation results. However, the results demonstrate some small deviations between the simulation and measurement. The measured resonant frequencies are shifted a little from the simulation and the insertion loss magnitude is lower than simulated one. This is because of mismatch between the feed-lines and SMA connectors and also the tolerance of fabrications which limits in simulation accuracy.

Previous several studies have demonstrated the advantages of planar resonator sensors with their compactness in circuit size and ease of fabrication. However, the planar techniques suffer from low sensitivity with poor Q-factor which restrict their use and limit their capabilities of detecting small changes in dielectric properties of materials [16]. Table 2 gives a clear picture of the comparison between the performance of the proposed RF sensors and the performance of other RF sensors. The proposed sensors show higher sensitivity with low insertion loss compared with other RF sensors from [16–20]. Furthermore, the SSRR sensor with double spurlines has the highest Q-factor value peaked up to 652.94 at 2.22 GHz resonant frequency when comparing to normal SSRR and SSRR with single spurline filter. This indicates that the compact SSRR with double spurlines has more efficiency and sensitivity compared to other RF sensors, even though the RF sensor in [19] has the lowest insertion loss. The circuit size of the proposed SSRR sensors have been minimized by about 30% compared to normal SSRR and achieved a high-quality factor (Q) for high sensitivity, accuracy, and simplicity of analysis. The small size means a high fraction of electric field energy will be penetrated in the sample, so the resonator sensor will be more sensitive to the presence of the sample.

**Table 2:** Comparison between normal SSRR, single and double spurlines filters.

Specifications	Proposed SSRR sensors			[17]	[18]	[16]	[19]	[20]	
	Normal SSRR [21]	with Single Spurline	with Double Spurlines						
Frequency [GHz]	2.22	2.22	2.22	2.57	1.28	2.65	2.44	10.66	10.95
Q-Factor	407.34	267.07	652.94	≈ 124	≈ 64	80	147	52	56
S21 [dB]	-15.32	-14.55	-8.92	-16.60	-22.34	-21.00	-5.52	-18.50	-18.50

**Fig 11:**
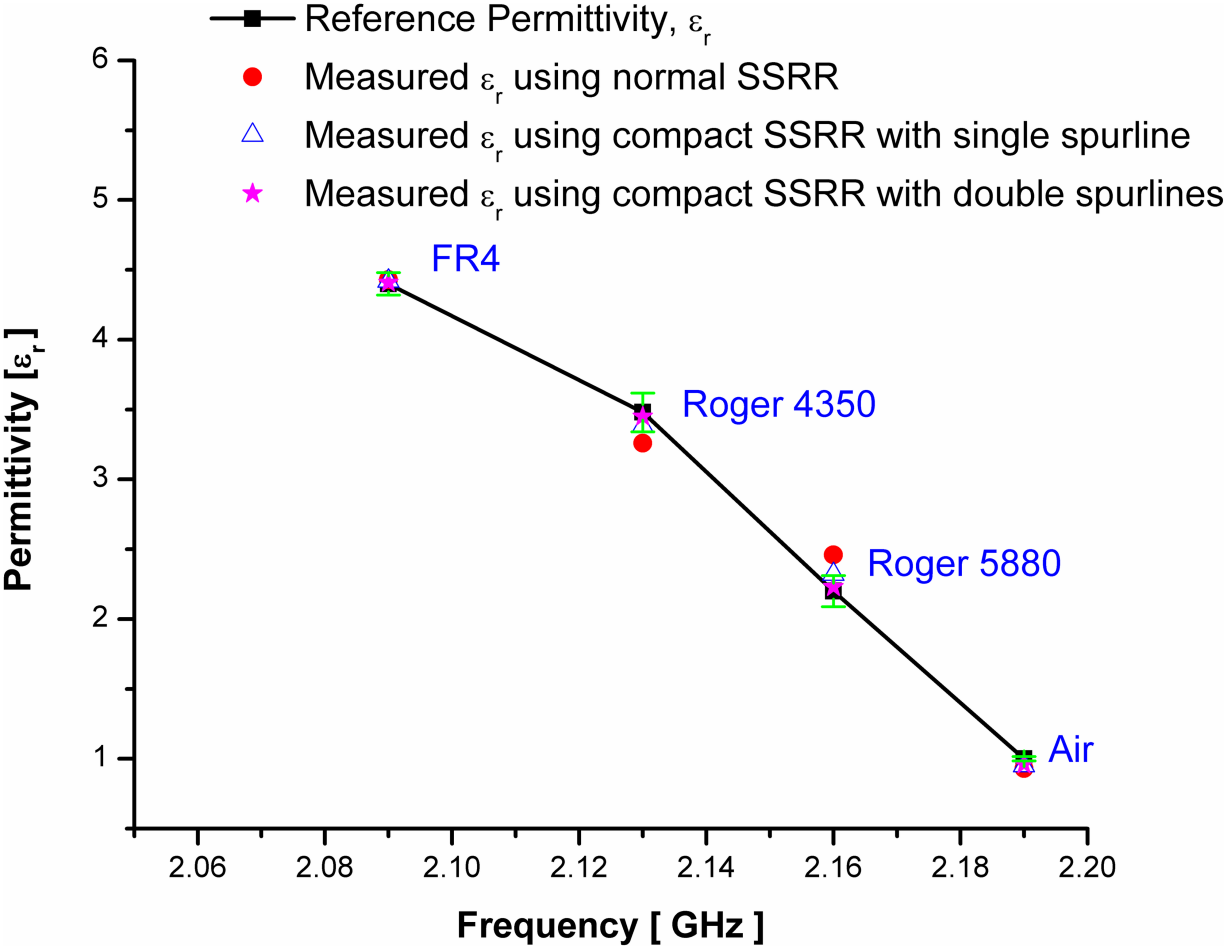
The measured permittivity of the proposed sensors in comparing with the standards permittivity of MUT; the bars in green color represents the standard deviations.

To validate the proposed sensors, practical standard materials with known permittivity are tested. The changes in measured resonant frequency are analyzed and accordingly, the mathematical equation is driven to extract the materials under test (MUT) properties. The measured permittivity corresponding to the resonant frequency of the standard material under test for the proposed sensors is indicated in Fig 11. The graph clearly illustrates the accuracy and sensitivity of the proposed sensors when compared the measured permittivity using these sensors to those found in the literature. To ensure the accuracy and repeatability of the measurement, experimental results were repeated 10 times at a room temperature at 25°C which allows the measurement of the maximum deviation from a known MUT over time. Then, the obtained data for each measurement was processed to obtain the resonance frequency and its mean value with respective standard deviation. The results shown in Fig 11 with a green color has a high repeatability of the sensor and small standard deviation for four experimental cases. The maximum standard deviation value of 0.13845 is exhibited by using SSRR with spurline at 10 times measurement for Roger 4350 material which is very small and close to the mean value of the resonance frequency. As a conclusion, the proposed devices have a great reliability to detect a change in the dielectric permittivity of different samples. Table 3 describes the permittivity of the tested standard materials using the proposed sensors and compared with their standards permittivity which is found in literature [22,18,23]. It can be clearly seen that the compact SSRR with double spurlines has the highest sensitivity with a typical average error of 2 to 3% compared to the normal SSRR [as indicated in [21] and compact SSRR with single spurline sensors. Both single and double spurlines sensors are designed by the integration of symmetrical split ring resonator [21] and the band-stop spurline filters for harmonic suppression. This advantage makes the proposed sensors more suitable for microwave devices by compactness in circuit size and high Q sensitivity and percentage of error as demonstrated in Table 3. Furthermore, the circuit size of SSRR sensor [21] has been minimized by about 30% of the total size by introducing spurline filters.

**Table 3:** Overall summary of experimental results for the SSRR sensors with/without spurline filters.

MUT	Reference Permittivity	Estimate error percentage (%) of Measured Permittivity using					
		normal SSRR [21]		SSRR with single spurline		SSRR with double spurlines	
		*ε*_*r*_	*% ε*_*r*_	*ε*_*r*_	*% ε*_*r*_	*ε*_*r*_	*% ε*_*r*_
Air	1	0.94	6.00	0.95	5.00	0.96	4.00
Roger RT 5880	2.2	2.12	3.63	2.32	5.45	2.23	1.37
Roger RT 4350	3.48	3.5	0.57	3.38	2.87	3.45	0.87
FR4	4.4	4.45	1.13	4.42	0.45	4.41	0.23

**Fig 12:**
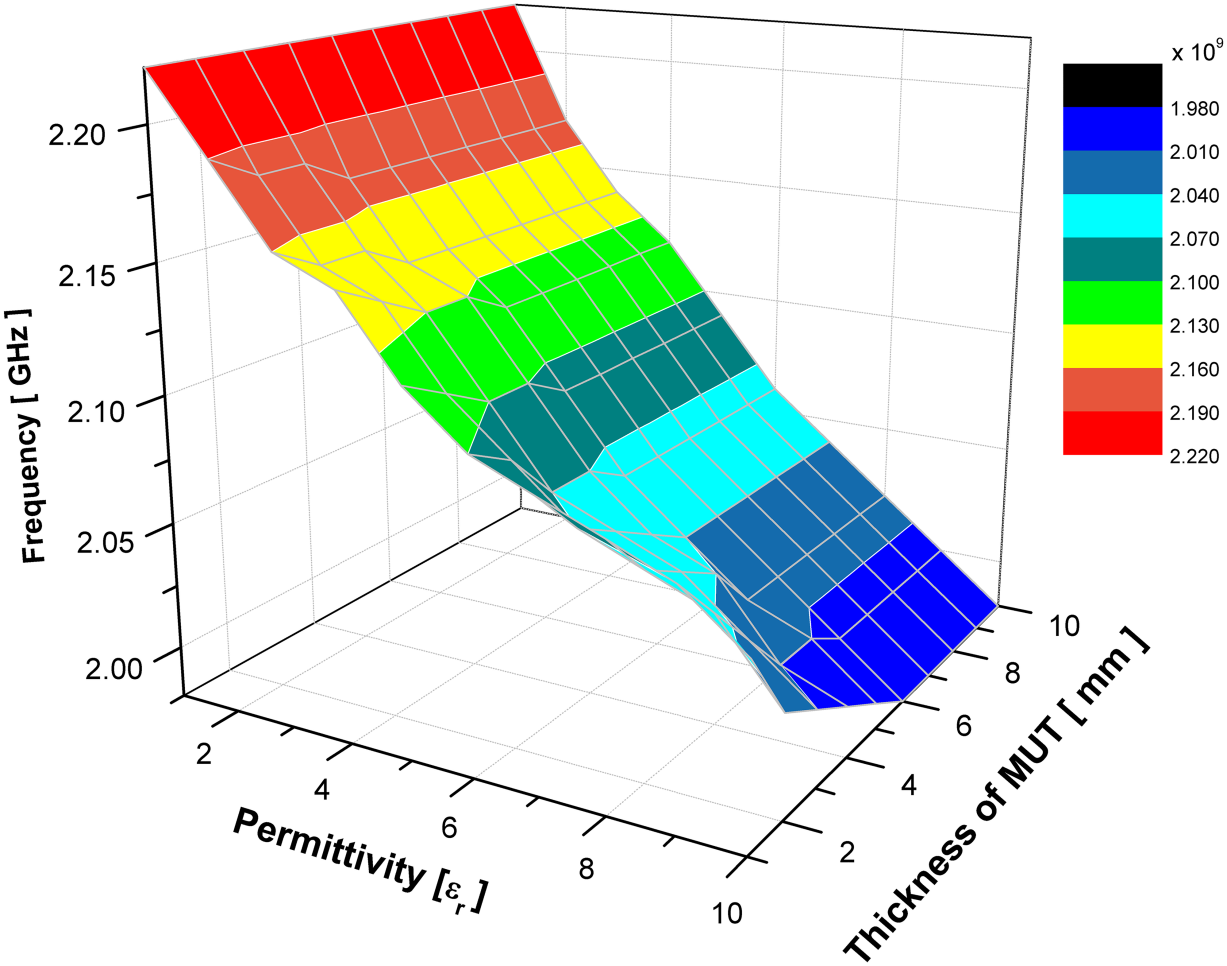
Effects of the resonance frequency with respect to change various value of sample’s real permittivity and thickness for SSRR sensor with single spurline.

**Fig 13:**
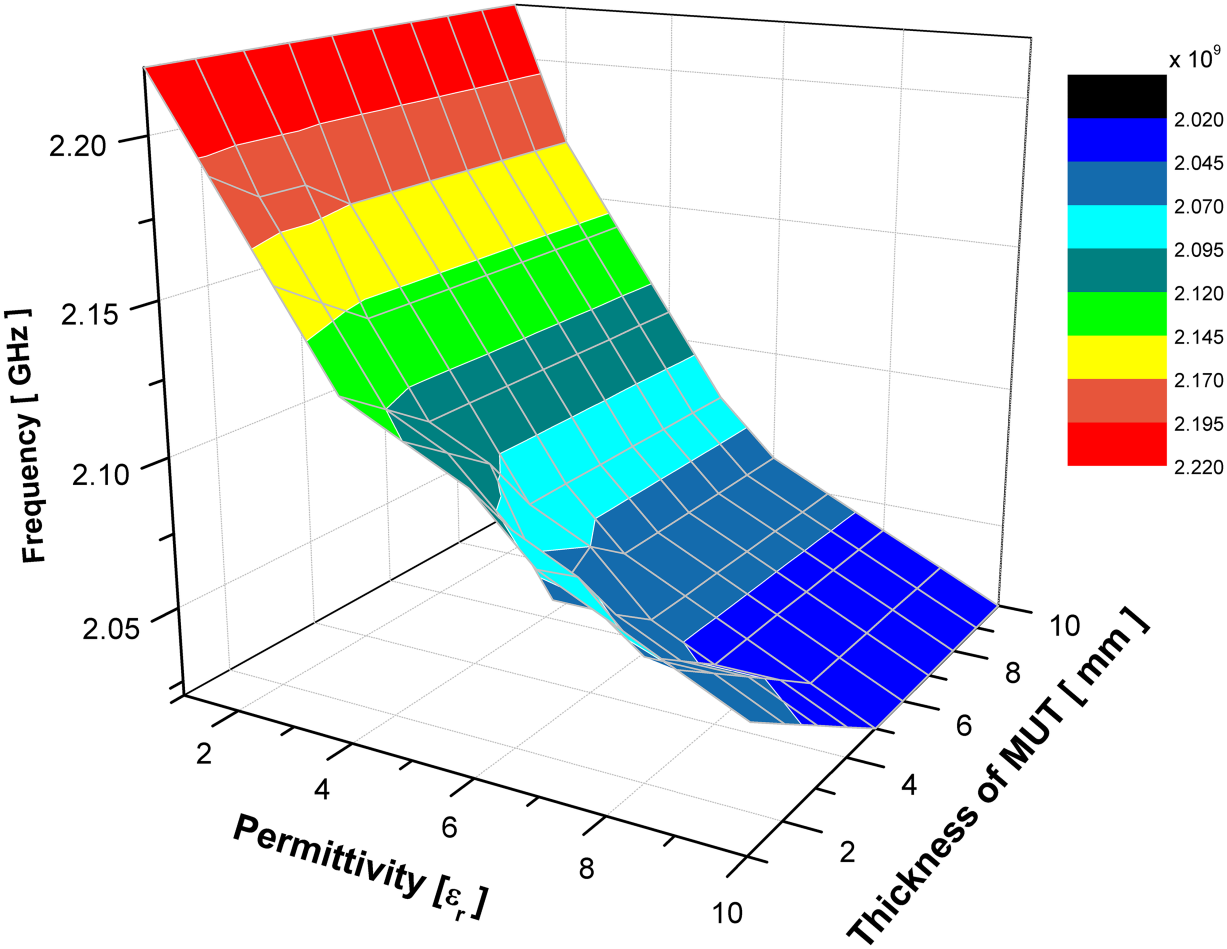
Effects of the resonance frequency with respect to change various value of sample’s real permittivity and thickness for SSRR sensor with double spurlines.

For the limit of detection, an estimation of 5 mm or more as a minimum amount of a sample’s thickness for tested materials that an analytical sensor’s process can reliably detect. The analyte can be present at lower concertation of sample’s thickness below the value given, however for more accuracy and reliability the detection limits are used as reporting limits for the proposed sensors. Figs 12 and 13 illustrate the change of MUT real permittivity and thickness in respect to the resonant frequency which is extracted from the simulated result of SSRR with single spurline and double spurlines, respectively. It can be clearly seen that the slope of plotted curve is dependent on the MUT thickness and it remains constant when the sample thickness is more or equal 6 mm for SSRR with single spurline and is more or equal 5 mm for SSRR with double spurlines. This happens due to the fact of increasing the overlaying sample, the shift in resonance frequency will be increased. A range of sample’s thickness is analyzed to find the point where further increment will not affect the resonant frequency. Therefore, a further increment of thickness size will not affect the resonance frequency effectively. This is due to the increment of the effective permittivity until it reaches the asymptotic value which demonstrates the electric field penetrated into the overlay sample. The result is in the lines of earlier literature [24] that found the saturation point is about 5% of MUT thickness.

**Fig 14:**
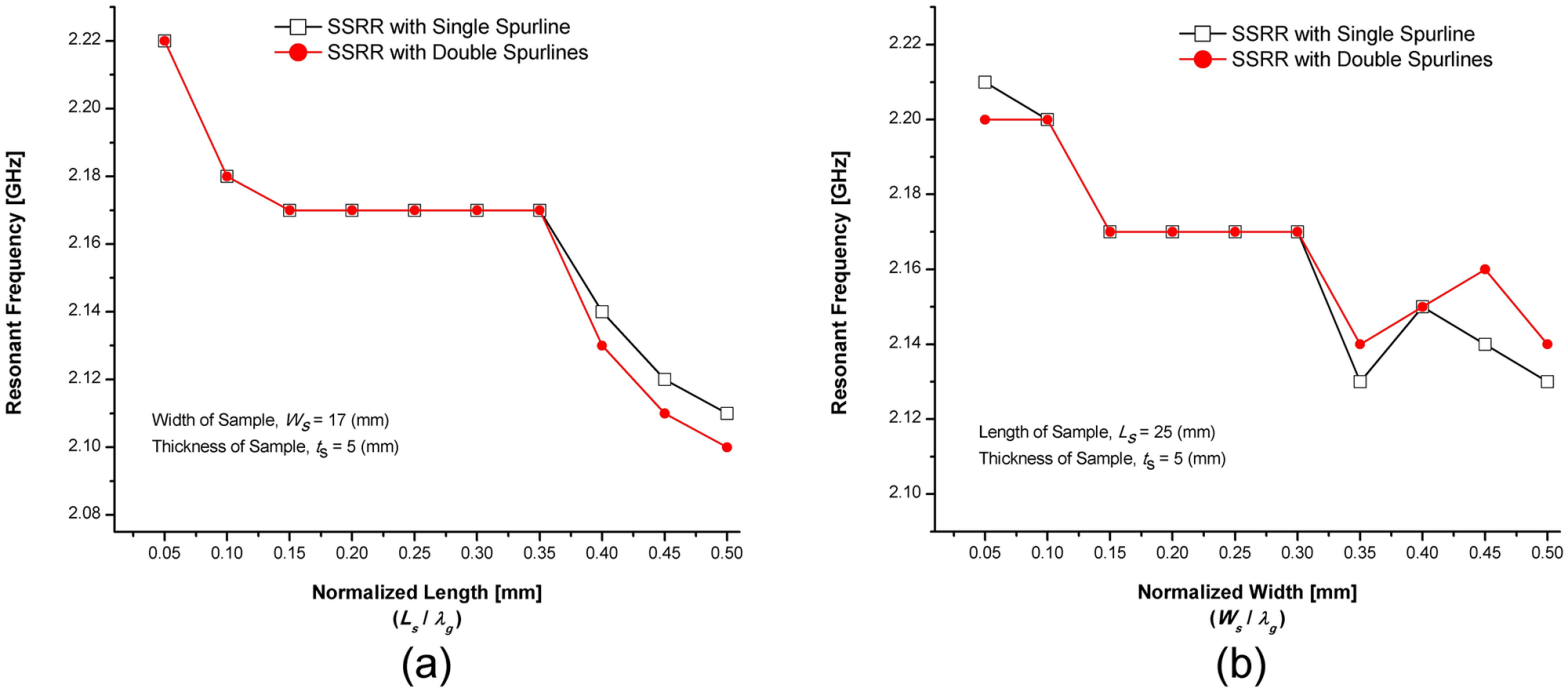
Effects of the sample size in resonant frequency (a) varying length with respect to fixed width and thickness (b) varying width with respect to constant length and thickness.

A numerical study was conducted in order to evaluate the sensitivity of the sensors where the length of the sample was varied over the range 1–50 mm (with an interval of 5 mm) while keeping the sample width and thickness fixed at 17 mm and 5 mm, respectively. Fig 14(a) demonstrates the effect of sample size and resonant frequency by varying the normalized length for fixed width and thickness. By normalizing the sample length (*L*_*s*_) with respect to the waveguide length (*λ*_*g*_), It can be observed that the resonance frequency decreases when the sample length is increased. Up to 15% of the resonator (As the corresponding diameter of 2.2 GHz ring resonator is about 31.8% of *λ*_*g*_) shows a continuous decrease in resonance frequency. While a range between 15–35% of *λ*_*g*_ demonstrates a constant resonance frequency which is considered as saturation points. Then, from 35% to 50% of *λ*_*g*_ illustrates continuous decreases in resonance frequency. Another study was conducted where the sample width varied over the range 1–50 mm (with an interval of 5 mm) while keeping the sample length and thickness constant at 25 mm and 5 mm, respectively. The change of resonant frequency and sample size by varying the normalized width with respect to a constant length and thickness is illustrated in Fig 14(b). For a normalized width (*W*_*s*_), up to 15% of waveguide length (*λ*_*g*_) shows a continuous decrease in resonance frequency similar to the effect of length. Then, from 15% to 30% of waveguide wavelength (*λ*_*g*_) illustrates a saturation point since the area over the ring sensor covered the maximum electric field distributions. After 30% of waveguide wavelength (*λ*_*g*_), the effect on resonance frequency becomes relatively small.

### Resonant frequency analysis

**Fig 15:**
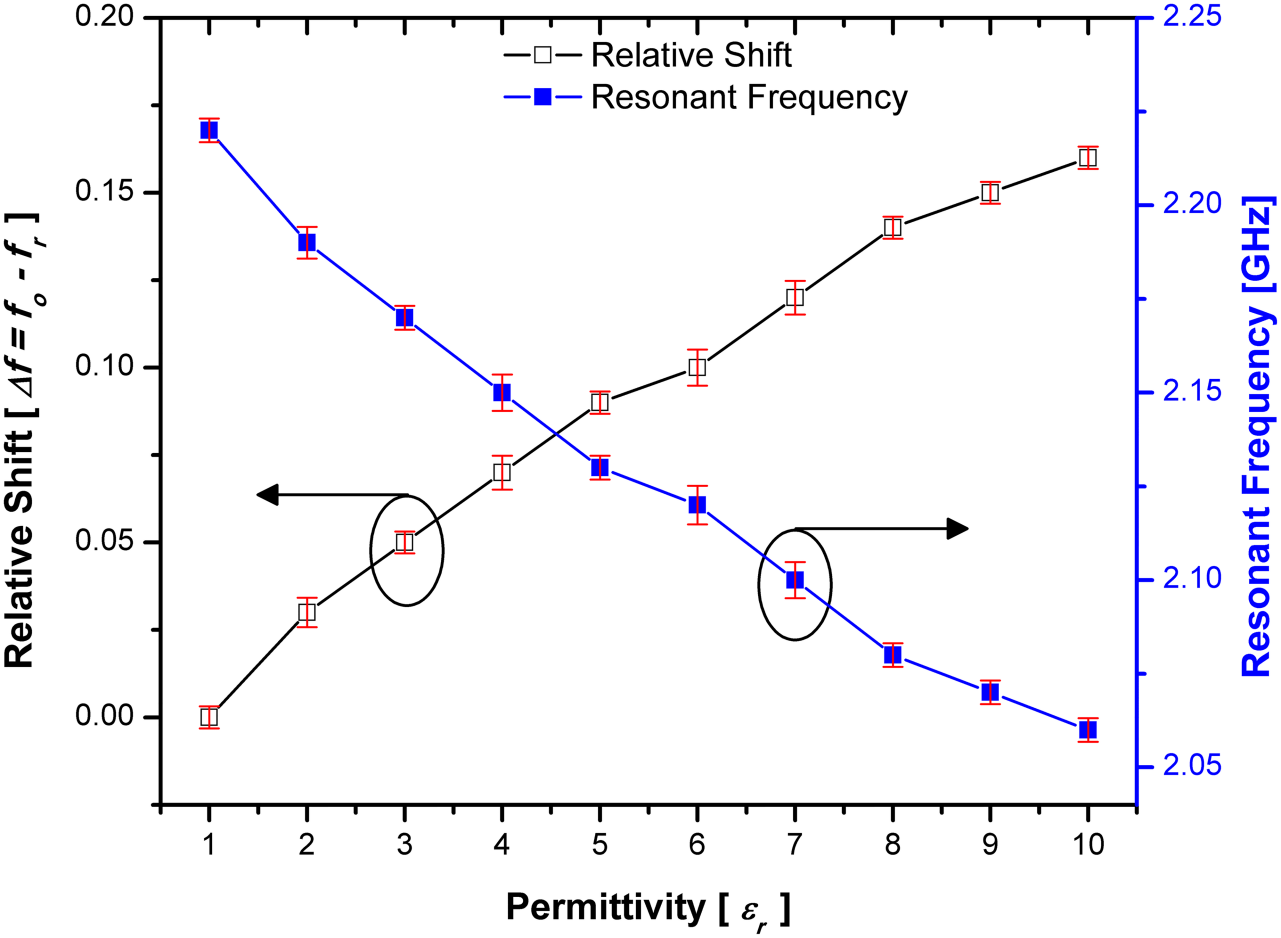
Sensitivity response in terms of relative shift and resonance frequency corresponding to various MUT permittivity for SSRR sensor with single spurline and the red bars are representative of the standard deviations.

**Fig 16:**
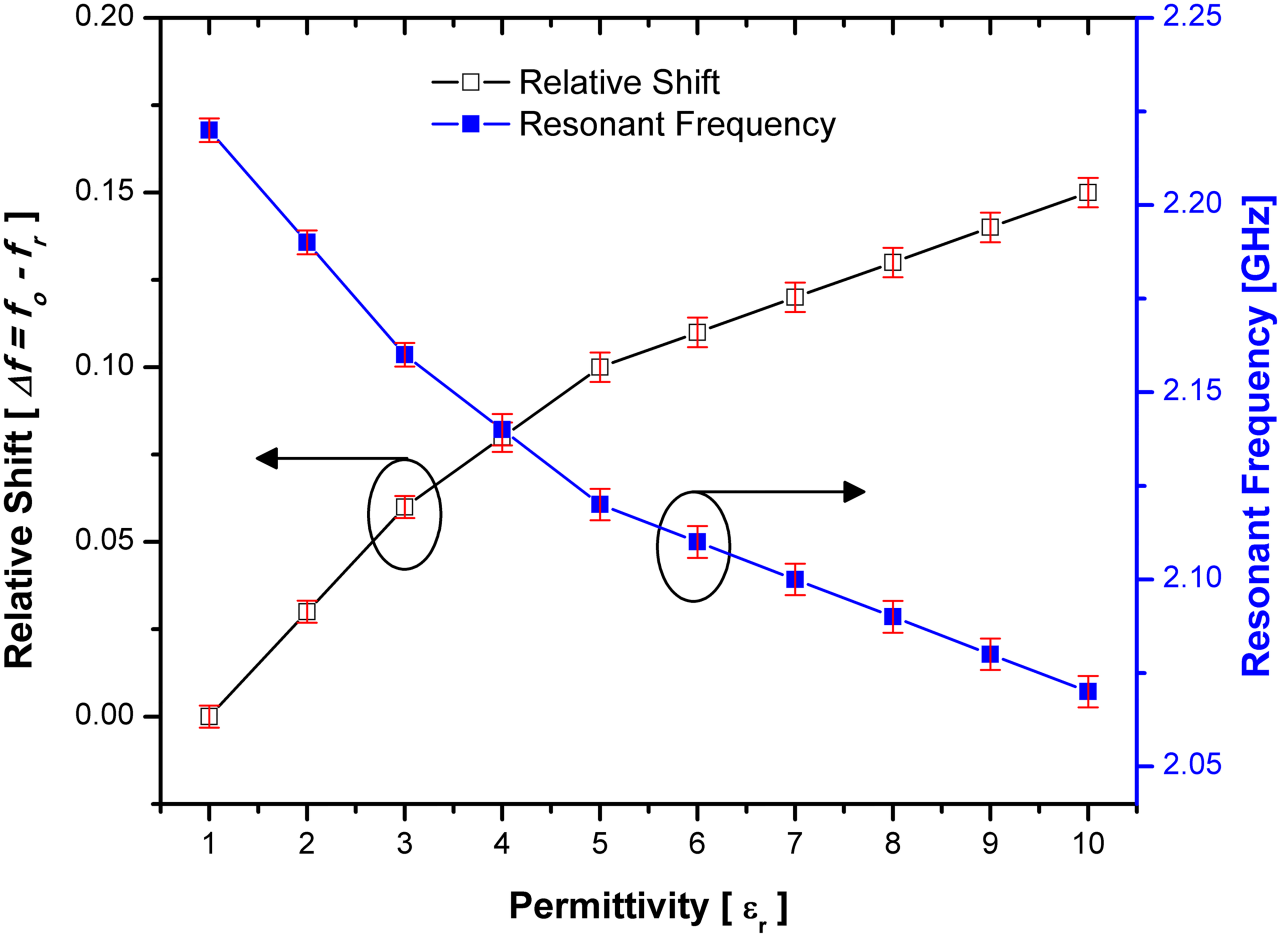
Sensitivity response in terms of relative shift and resonance frequency corresponding to various MUT permittivity for SSRR sensor with double spurlines and the red bars are representative of the standard deviations.

The interaction between the material under test (MUT) and the electric field of the designed sensors causes a change in resonance frequency and Q-factor. The change of resonant frequency and relative shift based on the variation of the material under test (MUT) is demonstrated in Figs 15 and 16 for both sensor designs with single and double spurlines filters respectively. The relative shift indicates the sensitivity of SSRR with single spurline. Where the highest shift occurs, the lower sensitivity produced. Unlike SSRR sensor, the SSRR with single spurline achieves high sensitivity since its relative shift is around 0.16 compared to SSRR sensor at same permittivity value of 10. This makes it a good choice for high sensitivity material characterization with the capability of detecting various materials at a small range of frequencies. The red bar in both Figs 15 and 16 are representative of the standards deviations which are considered very small standard deviations. Furthermore, the resonant frequency is shifted to 2.06 GHz compared to the SSRR sensor which is shifted to 2.01 GHz for a material permittivity of value 10. The resonant frequency is dependent on the properties of dielectric materials. However, the resonant frequency is shifted from 2.2 GHz resonant frequency at permittivity value of 1 to 2.07 GHz resonant frequency at permittivity value of 10 in the double spurlines. This indicates that the SSRR with double spurlines achieved the lower shift compared to the normal SSRR and SSRR sensor with single spurline. Moreover, the sensitivity of the SSRR sensor with double spurlines can be indicated by relative shift where the lower shift occurs, the highest sensitivity produces. Unlike the normal SSRR and SSRR sensor with single spurline, the SSRR with double spurlines achieves the highest sensitivity since its relative shift is around 0.15 compared to the normal SSRR and SSRR with single spurline at the same permittivity value of 10. Therefore, the SSRR sensor with double spurlines can characterize a small change in the dielectric permittivity of the measured MUT with a low insertion loss. It is anticipated that this sensor could be effectively used to study the properties of materials with a variation of the permittivity value in greater detail than can be obtained from the normal SSRR sensor and SSRR sensor with single spurline techniques.

### The Q-factor analysis

**Fig 17:**
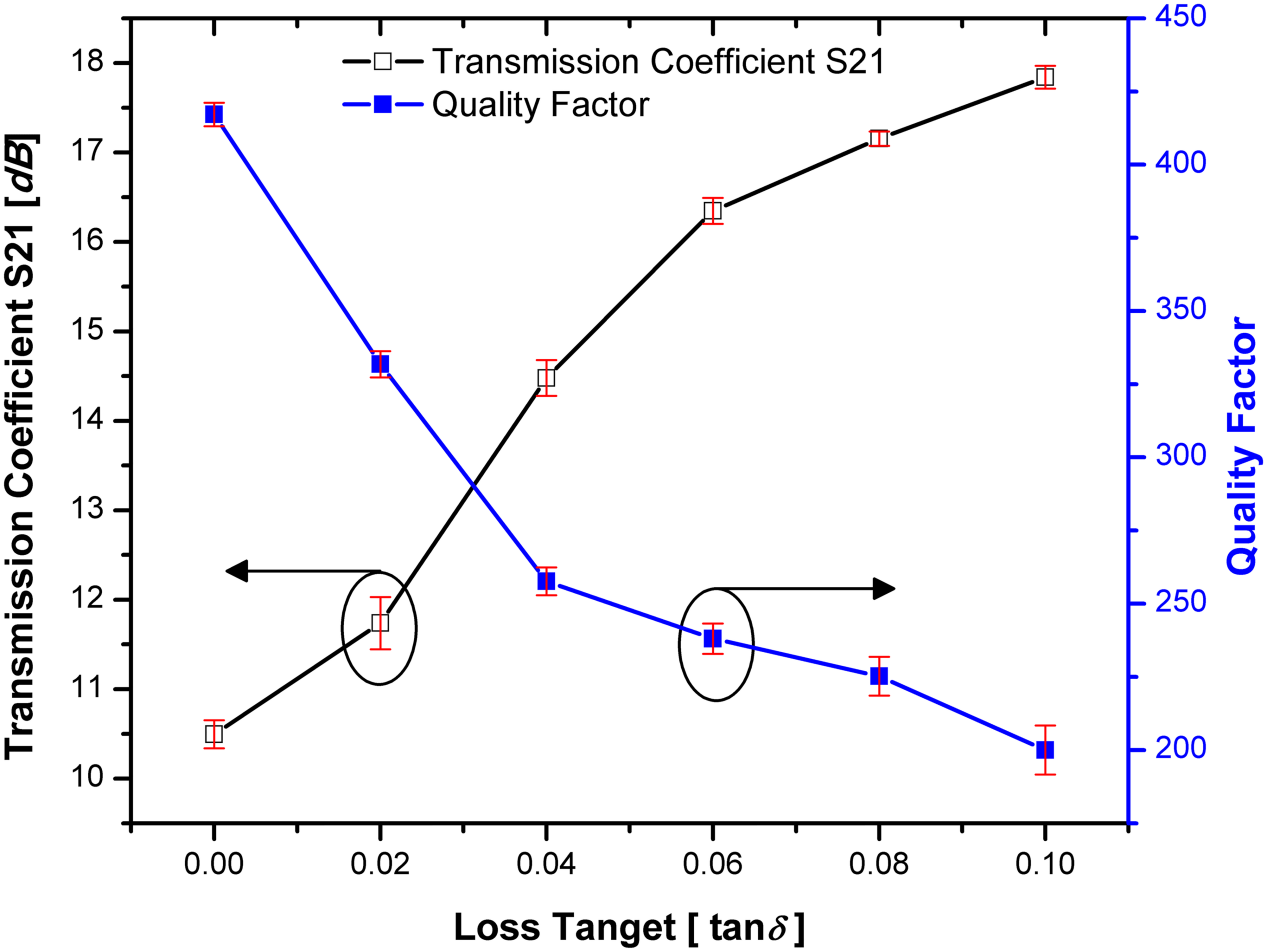
The change of quality factor and transmission when changing the MUT loss tangent for SSRR sensor with single spurline and the red bars are representative of the standard deviations.

**Fig 18:**
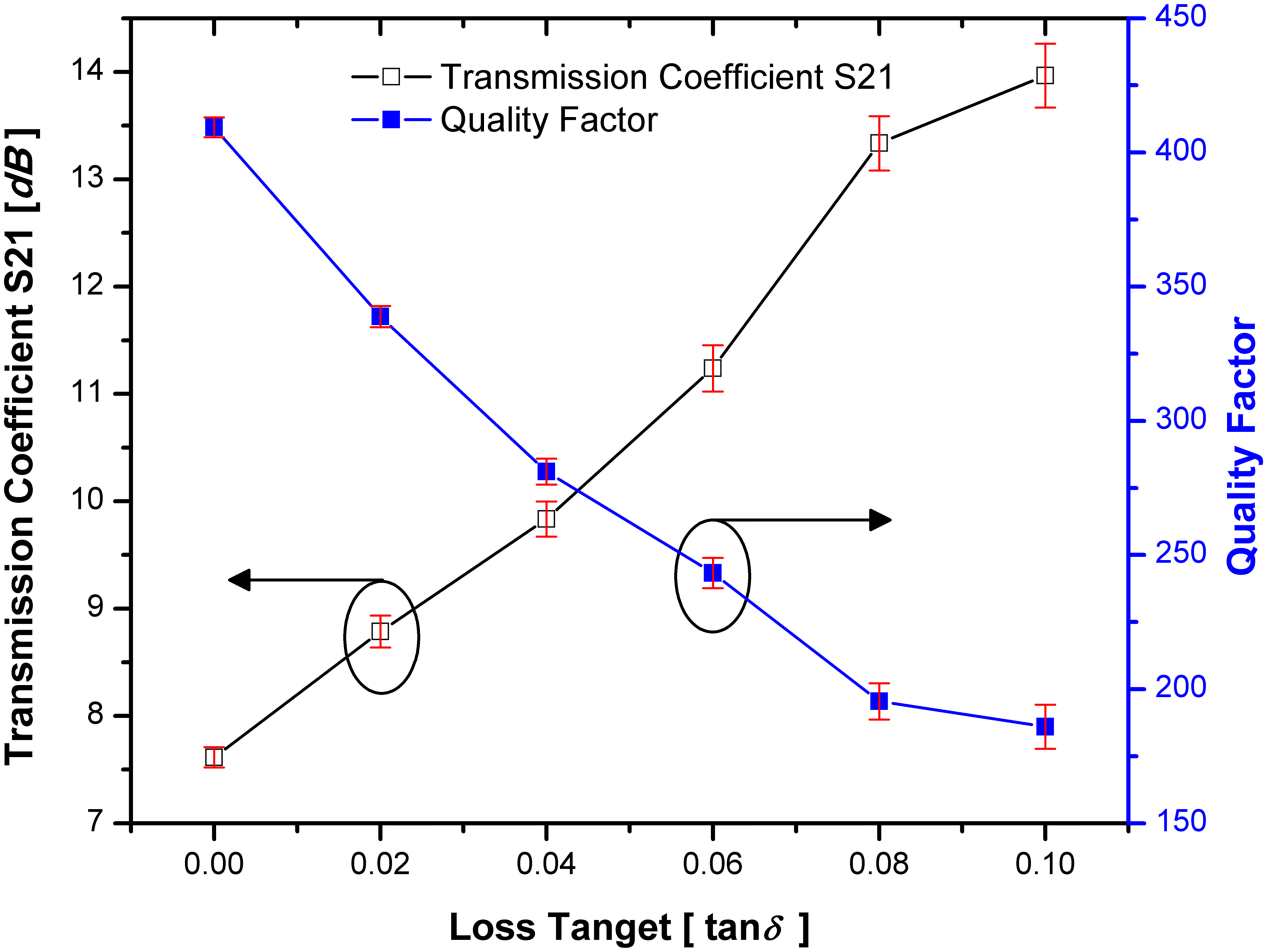
The change of quality factor and transmission when changing the MUT loss tangent for SSRR sensor with double spurlines and the red bars are representative of the standard deviations.

By considering the permittivity value of tested material to 2 (*ε*_*r*_ = 2), Fig 17 demonstrates the quality factor and transmission coefficients when changing the tangential loss of tested materials for SSRR with single spurline. It can be seen that the transmission coefficient is directly proportional to loss tangent of the tested material while the quality factor is inversely proportional to the loss tangent of the tested materials which is decreased to 267.07 at 2.22 GHz resonant frequency. Due to the high sensitivity of the SSRR sensor with double spurlines, it can characterize small changes in the dielectric permittivity of the measured material under test (MUT). The loss tangent (tan δ) is taken into consideration due to its effects on the quality factor of the SSRR sensor with double spurlines. Fig ‎18 illustrates the change in the loss tangent of the MUT in a range of 0 to 0.1 corresponding to the quality factor and transmission coefficient S21 (*dB*) where the tested material has a permittivity of 2 value (*ε*_*r*_ = 2). It is quite interesting to note that the sensitivity of the sensor is dependent on the change in the tangential loss of the tested material where an increasing the loss tangent of tested material leads to a decrement of the quality factor value and increment of transmission coefficients. The red bars in Figs 17 and 18 are representative of the standard deviations and it can be concluded that when the loss tangent of tested materials increased, the value of standard deviation will be increased.

## Conclusion

In this paper, we have presented for the first time the use of symmetrical split ring resonators (SSRR) sensors with spurline filters for the properties of materials characterizations. These techniques offer several advantages over current methods in that they utilize the compactness of a planar device and high-quality factor with high sensitivity. As a result of the SSRR sensor with spurline filters, accurate measurements on solid samples can be performed. The use of planar perturbation method in evaluating the permittivity from the measured transmission coefficient offers simplicity and accuracy. For demonstration purposes, a symmetrical split ring resonator with spurline filters resonating at 2.2 GHz has been designed and fabricated in Roger RT 5880 material with low loss substrate. The introducing of single and double spurlines filter is to reject undesired harmonic frequency. Thus, the sensitivity and quality factors are enhanced which can detect and characterize the material properties with a small variation of permittivity. Furthermore, the normal size of the symmetrical split ring resonator (SSRR) is minimized by about 30% of the total size when introducing the spurline filters. Therefore, the proposed sensor device performs a high Q-resonator with high sensitivity and accurate measurements of characterizing the properties of materials and low cost due to its compactness in size. The measurements are compared to each used method and the percentage error in the measured results in each case is within ±1.3%. The ease of fabrication and low cost of manufacture makes these type of sensors ideal for use in many industrial applications such as food industry, quality control, biomedical and therapeutic goods.

## Supporting information

S1 Dataset Relevant data for Fig 8. (DAT)

S2 Dataset Relevant data for Fig 9. (DAT)

S3 Dataset Relevant data for Fig 10. (DAT)

S4 Dataset Relevant data for Fig 11. (DAT)

S5 Dataset Relevant data for Fig 12. (DAT)

S6 Dataset Relevant data for Fig 13. (DAT)

S7 Dataset A. Relevant data for Fig 14A. B. Relevant data for Fig 14B.
(DAT)

S8 Dataset Relevant data for Fig 15. (DAT)

S9 Dataset Relevant data for Fig 16. (DAT)

S10 Dataset Relevant data for Fig 17. (DAT)

S11 Dataset Relevant data for Fig 18. (DAT)
